# Benefit–Risk Perceptions and Public GenAI Acceptance: A Survey Experiment Based on the Emerging Technology Acceptance Model

**DOI:** 10.3390/bs16050732

**Published:** 2026-05-09

**Authors:** Ning Ma, Songtao Lin, Xinyu Dong, Hao Ji

**Affiliations:** School of Public Policy and Administration, Xi’an Jiaotong University, Xi’an 710049, China; maning@xjtu.edu.cn (N.M.); songtaolin@stu.xjtu.edu.cn (S.L.); 964355352@stu.xjtu.edu.cn (H.J.)

**Keywords:** generative artificial intelligence, Technology Acceptance Model, risk perception, benefit perception, survey experiments

## Abstract

Drawing on the Emerging Technology Acceptance Model, this study integrates perceived benefits and perceived risks into a unified analytical framework to examine public acceptance of generative artificial intelligence (GenAI). Using three scenario-based survey experiments, the study shows that perceived usefulness and perceived ease of use significantly promote GenAI acceptance, whereas perceived social welfare loss, personal property loss, and technical–ethical conflict all significantly reduce acceptance. The negative effects of these three risk scenarios also differ in magnitude, with personal property loss exerting the strongest inhibitory effect. Heterogeneity analyses further indicate that technology usage behavior moderates these relationships selectively rather than universally, with stronger evidence on the risk side than on the benefit side. In addition, lower confidence in regulatory oversight is more clearly associated with stronger risk sensitivity, whereas evidence for stronger benefit sensitivity under higher regulatory trust remains weak. These findings deepen understanding of the differentiated benefit–risk trade-offs shaping attitudes toward emerging AI technologies among Chinese internet users in an online survey context.

## 1. Introduction

Artificial Intelligence (AI) was first coined at the Dartmouth Conference held in the summer of 1956, marking the birth of the discipline of artificial intelligence. Entering the 21st century, with the rapid advancement of generative AI technologies represented by Large Language Models (LLMs), their societal impact has increasingly drawn widespread attention (Xie & Avila, 2025). Large language models typically refer to ultra-large parameter models that, through pre-training on vast corpora, can capture contextual semantics and generate natural language text (Yao et al., 2024). Currently, a variety of large language models have been developed worldwide, including GPT-4o and DeepSeek-R1, which are widely applied in healthcare (Thirunavukarasu et al., 2023), code generation, cybersecurity and other fields, demonstrating formidable cross-industry language processing capabilities and technological potential.

In early 2025, the DeepSeek-R1 model was released. With performance rivalling GPT-4o, significantly reduced training costs, and its open-source nature, it achieved a major breakthrough in large language models. As large models enter the era of open-source development and large-scale deployment, the gap between technology and the public has narrowed further. Generative Artificial Intelligence (GenAI) is progressively becoming a tool accessible to the general public in their daily lives. Much like the steam engine reshaped societal structures during the Industrial Revolution, GenAI similarly demonstrates profound transformative potential and innovative capabilities across diverse domains, while broader AI applications have also been linked to green growth and sustainable development (Zhao et al., 2025). As GenAI rapidly moves from expert-oriented development to mass adoption, understanding how the public evaluates its benefits and risks becomes increasingly important for both technology diffusion and AI governance.

Despite significant advances in GenAI technology across multiple domains, as a quintessential emerging technology, its technical trajectory remains immature and its dominant paradigm is still taking shape. With the escalating capabilities of models and the expansion of large-scale applications, AI governance faces increasingly complex challenges encompassing multiple issues such as technological uncertainty, societal institutional adaptability, and ethical risks (Gasser & Almeida, 2017). Related research on digital technologies also suggests that insufficient policy frameworks may become a barrier to sustainability-oriented innovation and broader technology diffusion (Watts et al., 2025). Therefore, conducting an in-depth exploration of public GenAI acceptance and its underlying mechanisms holds significant importance for promoting the healthy dissemination of the technology, refining AI governance frameworks, and advancing its societal applications.

Emerging Technologies typically refer to technological forms that are still in their early stages of development or practical application. Such technologies not only possess the potential to transform the status quo, but also present both opportunities and challenges for national technological strategy (Cozzens et al., 2010). It is widely recognised within academic circles that emerging technologies exhibit the following defining characteristics: radical novelty, relatively rapid growth, coherence, significant impact, and a high degree of uncertainty and ambiguity (Rotolo et al., 2015). Generative AI demonstrates extensive societal influence throughout its technological evolution, whilst simultaneously exhibiting high levels of uncertainty and ambiguity. This aligns with the fundamental characteristics of emerging technologies, presenting a dual nature where benefits coexist with risks.

In terms of benefits, GenAI has demonstrated significant value in areas such as data governance, text classification, and sentiment analysis (Gilardi et al., 2023; Brown et al., 2020). Its robust language comprehension and generation capabilities (Chang et al., 2024) can substantially enhance work efficiency and decision-making quality, thereby increasing public acceptance of the technology. Generative AI can significantly enhance work efficiency and decision-making quality across daily office operations, government affairs, education, and research, while providing intelligent assistance in specialised fields such as healthcare, finance, law, sustainability assessment (Chen et al., 2025), and operations management (Khlie et al., 2024). With the continuous advancement of technology, GenAI’s learning and application capabilities across multiple domains continue to strengthen. It has already achieved human-level performance, or even surpassed it, in certain tasks, further driving its widespread adoption across diverse scenarios.

At the same time, the risk attributes of GenAI must not be overlooked. Potential issues including fairness, impartiality, lack of accountability mechanisms, data misuse, and insufficient model robustness may all provoke public resistance to the technology. This could adversely affect its acceptance, thereby constraining its widespread adoption and development (Zhu & Lou, 2024). Existing research typically categorises the risks of GenAI into three types: firstly, the direct risks that have already attracted widespread attention; secondly, systemic new issues that may arise as the technology expands; and thirdly, potential catastrophic and existential risks that could have profound implications for human society (Guan et al., 2024; Mollen, 2024; Yao et al., 2024).

In summary, although existing literature has explored the societal impacts of GenAI from various perspectives, much of it tends to focus primarily either on its benefits and application value (e.g., Brown et al., 2020; Chang et al., 2024; Gilardi et al., 2023) or on its risks, ethical concerns, and governance challenges (e.g., Gasser & Almeida, 2017; Haltaufderheide & Ranisch, 2024; Yao et al., 2024), leaving limited work that integrates both sides into a unified analytical framework. Existing studies have also paid relatively limited attention to how benefit–risk evaluations may vary across different risk scenarios and under different conditions of technology usage behavior and regulatory trust. To address these gaps, this study aims to explain public acceptance of GenAI by integrating perceived benefits and perceived risks into a unified analytical framework and by examining how this relationship varies across distinct risk scenarios, technology usage behavior, and regulatory trust.

Theoretically, this study extends the classic Technology Acceptance Model by incorporating risk perceptions associated with emerging technologies and by introducing individual usage behavior, group usage behavior, and trust in regulation as moderating factors. Based on this benefit–risk framework, it further distinguishes among social welfare loss, personal property loss, and technical–ethical conflict in order to examine whether different types of perceived risk shape public acceptance through different mechanisms. Methodologically, this study adopts a survey-experimental design with three context-specific scenario interventions, which helps reduce some common-source bias inherent in traditional questionnaire surveys and allows clearer identification of attitudinal responses to different types of risk information under randomized exposure. Practically, the study highlights the importance of understanding public responses to GenAI in context and provides empirical evidence for policy risk communication and public discussion of AI governance.

The remainder of this paper is organized as follows. Section 2 presents the hypotheses and research model of this study. Section 3 describes the research design and data. Section 4 reports the empirical results. Section 5 concludes the study and discusses its limitations and directions for future research.

## 2. Hypotheses and Research Model

### 2.1. Hypotheses

#### 2.1.1. Benefits Perception

Based on rational behavior theory, Davis (1989) proposed the Technology Acceptance Model (TAM) in 1989 to explain low information system usage rates. This model posits that an individual’s willingness to adopt technology is primarily influenced by two factors: perceived usefulness and perceived ease of use. These two dimensions have been repeatedly validated as core determinants of technology adoption in subsequent TAM-related research (Marangunić & Granić, 2015; Venkatesh et al., 2003). This logic also remains relevant in other emerging digital-technology contexts, where perceived usefulness and perceived ease of use continue to shape users’ willingness to adopt complex technological systems (Jebbor et al., 2025). Perceived usefulness refers to an individual’s subjective judgement that employing a particular technology will enhance their work performance; Perceived ease of use denotes the degree to which an individual believes using the technology requires minimal effort. Together, these factors determine an individual’s attitude towards technology adoption, subsequently influencing behavioral intentions and actual usage. Consequently, this study builds upon the traditional TAM’s perspective of perceived benefits to define the public’s perceived benefits of GenAI: namely, the subjective judgement that using GenAI yields benefits without requiring excessive effort. Based on the public’s perceived benefits of GenAI, the following hypotheses are proposed:

**H1.**  *Increased perceived usefulness of GenAI among the public will enhance public acceptance of the technology.*

**H2.**  *Increased perceived ease of use of GenAI among the public will enhance public acceptance of the technology.*

#### 2.1.2. Risk Perception

Risk perception is typically defined as individuals’ subjective anticipation of potentially serious negative consequences associated with a decision (Barach, 1969). Compared with TAM, which primarily emphasizes perceived usefulness and perceived ease of use, and UTAUT, which further incorporates social influence and facilitating conditions, the Emerging Technology Acceptance Model is particularly relevant for emerging technologies because it explicitly brings risk, uncertainty, and broader social consequences into the acceptance framework (Zhu & Lou, 2024). This extension is especially important in the context of GenAI, where perceived benefits coexist with concerns about social welfare, personal loss, and technological–ethical conflict. Drawing on Zhu and Lou’s (2024) Emerging Technology Acceptance Model, this study defines the public’s perception of risks associated with GenAI as the subjective expectation that the use of GenAI may lead to serious or negative consequences. Building on the framework proposed by Rotolo et al. (2015) for characterising emerging technologies—which highlights risks along three tiers (“systemic impacts”, “individual consequences”, and “conflicts with social norms”)—we examine public perceptions of GenAI-related risks across three dimensions: social welfare risks, personal property risks, and technical–ethical risks. These three dimensions were selected to capture the main levels at which GenAI-related risks may be perceived by the public, namely macro-level social consequences, direct individual losses, and norm-based ethical concerns. Specifically, perceptions of social welfare risks capture macro-level concerns about GenAI’s potential threats to collective welfare, public-resource allocation, and social stability. Perceptions of personal risks capture micro-level concerns about direct material and livelihood-related harms to individuals, such as job displacement, privacy breaches, data breaches and economic insecurity. By contrast, perceptions of technical–ethical risks capture normative concerns about whether GenAI is consistent with fairness, transparency, accountability, and broader social values. To sharpen the conceptual distinction among these dimensions, personal property risks in this study refer primarily to direct individual harms, whereas technical–ethical risks refer to norm-based concerns regarding how GenAI systems operate and whether their outputs align with social and ethical expectations. Accordingly, we operationalise these three dimensions as scenarios of “social welfare loss”, “personal property loss”, and “technical–ethical conflict”, and develop separate hypotheses for each.

Generative AI may disrupt societal structures and resource allocation, leading to shifts in occupational landscapes and exacerbating social inequalities (Xie & Avila, 2025). For example, the 2023 Writers Guild of America (WGA) strike reflected the impact of new technologies on the labour market. Moreover, the capitalisation of GenAI has given rise to technological oligarchies (Barros, 2025), further exacerbating social inequality. With technology becoming widely accessible across various domains, the high mobility of data and information has created latent interdependencies between seemingly independent sectors. Studies on technology acceptance, such as those examining ICT usage (Guner & Acarturk, 2020), reveal that public adoption is influenced by societal factors. This study therefore posits that when the public perceives GenAI as detrimental to social welfare, their acceptance of the technology diminishes. Consequently, this study identifies potential societal structural transformations, social inequalities, and information security risks associated with GenAI as core dimensions for measuring perceived social welfare risks. Based on this, the following research hypotheses are proposed:

**H3.**  *Perceived social welfare loss associated with GenAI reduces public GenAI acceptance.*

Beyond posing threats to society as a whole, GenAI may also generate direct losses at the individual level, thereby affecting public willingness to adopt the technology. Empirical studies on nuclear power technology (Gong et al., 2022), WebCT (Ngai et al., 2007), and genetically modified foods (Mandal & Paul, 2012) suggest that when technological deployment is perceived as harming individuals’ safety, property, health, or other direct personal interests, public acceptance tends to decline.

In the context of GenAI, such personal property loss may arise in several ways. First, the widespread adoption of GenAI may increase the risk of job displacement, thereby threatening individuals’ income stability and economic security. Second, the training and deployment of large models involve substantial amounts of personal data, which may expose individuals to privacy breaches, data misuse, and loss of control over personal information (Yao et al., 2024). Third, technological monopolization may concentrate opportunities and economic returns in the hands of a limited number of actors, indirectly undermining individuals’ material interests and personal economic prospects (Xie & Avila, 2025). In this study, therefore, personal property loss refers to direct losses borne by individuals in relation to their employment security, personal data and privacy, and personal economic interests, rather than to broader collective welfare concerns or norm-based ethical conflicts. Consequently, this study identifies job displacement, data breaches, and technology-driven concentration of personal economic disadvantage as the primary dimensions of perceived personal property loss associated with GenAI. Based on this, the following hypothesis is proposed:

**H4.**  *Perceived personal property loss associated with GenAI reduces public GenAI acceptance.*

Ethics is commonly understood as a set of socially recognized norms and codes of conduct that guide behavior (Hegel, 1991). However, the rapid development of emerging technologies has placed traditional human–machine relations under unprecedented strain (Dhoni & Kumar, 2023). In the case of GenAI, although its versatile functions can improve efficiency and expand access to information, they may also generate serious normative concerns regarding fairness, transparency, accountability, and value alignment. Existing studies have shown that GenAI may produce discriminatory outcomes, obscure decision-making processes, and create conflicts with widely accepted ethical expectations and social norms (Haltaufderheide & Ranisch, 2024; Yao et al., 2024). Related research further suggests that perceptions of fairness, transparency, and trust significantly shape willingness to adopt AI systems in practice (Yaseen & Al-Amarneh, 2025). Unlike social welfare loss, which concerns collective consequences at the societal level, or personal property loss, which refers to direct harm to individuals’ employment security, personal data rights, and material interests, technical–ethical conflict in this study refers specifically to concerns that GenAI may violate accepted principles of fairness, responsibility, and ethical legitimacy. In this sense, the core of technical–ethical risk lies not in direct material loss itself, but in whether the operation and outcomes of GenAI are perceived as normatively appropriate and socially acceptable. Consequently, this study identifies issues such as algorithmic discrimination, insufficient transparency, and conflicts with prevailing ethical principles as the primary dimensions of perceived technical–ethical risk associated with GenAI. Based on this, the following hypothesis is proposed:

**H5.**  *Perceived technical–ethical conflict associated with GenAI reduces public GenAI acceptance.*

#### 2.1.3. Modifying Factors

This study posits that during the public’s engagement with GenAI, their technological usage behaviors exert a moderating influence on perceptions of benefits and risks, thereby further shaping their acceptance of the technology. Specifically, these usage behaviors can be categorised as follows: firstly, individual usage behaviors, referring to an individual’s personal engagement with GenAI; secondly, group usage behaviors, denoting the prevalence of GenAI usage within the social environment surrounding the public, encompassing family members, friends, colleagues, and similar groups.

Firstly, from the perspective of individual technological usage behavior, Social Influence Theory posits that an individual’s attitudes and actions within social contexts are shaped not only by personal experience but also by the opinions of others, persuasive messages, and social norms (Cialdini & Goldstein, 2004; Venkatesh et al., 2003). For emerging technologies, the greater the frequency of personal use, the more direct experience individuals accumulate regarding the technology’s functionalities and potential risks in practice. This enhances their sensitivity to both benefits and risks, and influences their technological acceptance through processes of social comparison and normative identification. Secondly, from the perspective of group usage behavior, Diffusion of Innovations theory (Rogers et al., 2014) posits that technology adoption depends not only on the attributes of the innovation itself, but also on its dissemination and imitation processes within social networks (Wejnert, 2002). When an individual’s group exhibits high usage frequency, the diffusion effect within social networks and peer demonstration enhance familiarity with and trust in the technology. This facilitates recognition of its potential benefits while heightening sensitivity to risk information exposed during use. This social diffusion mechanism renders group usage behavior a significant external condition influencing individual perceptions and attitudes. Existing research indicates that social influence is a key factor affecting perceived usefulness and perceived ease of use (Lu et al., 2005). Based on the above analysis, this study proposes that the GenAI usage behavior of the public themselves or their surrounding groups will heighten their sensitivity to the technology’s potential benefits and risks, thereby further influencing their willingness to accept the technology. Consequently, the following hypothesis is proposed:

**H6a.**  *Individuals who frequently use a particular technology are more likely to recognise its potential benefits and risks, thereby exhibiting heightened sensitivity to perceptions of both, which in turn influences their acceptance of the technology.*

**H6b.**  *Social-circle GenAI usage frequency positively moderates the relationships between perceived benefits/risks and GenAI acceptance: the more frequently a person’s social circle uses GenAI, the stronger the effects of perceived benefits and risks on their GenAI acceptance.*

Moreover, the level of trust in regulators also plays a significant role in moderating the influence of perceived benefits and risks on technology acceptance. Existing research indicates that trust occupies a central position in both risk perception and technology adoption (Shivani, 2012). Studies on controversial and emerging technologies further show that institutional trust shapes both perceived risks and perceived benefits, and that lower trust is often associated with stronger perceptions of risk and lower acceptance of new technologies (Siegrist, 2000; Siegrist & Cvetkovich, 2000). Slovic (1993) noted that public acceptance of risk is often not based solely on technical assessments, but is significantly influenced by the degree of trust in regulatory bodies and governance systems. Research on risk regulation also suggests that trust in regulatory institutions is especially important under conditions of uncertainty, because it affects whether the public regards a technology as controllable, fairly governed, and socially acceptable (Poortinga & Pidgeon, 2003). High trust mitigates the public’s perception of risk uncertainty. In other words, regulatory trust reduces individuals’ cognitive load, making them more inclined to adopt decisions based on simplified judgement pathways. Groups exhibiting high regulatory trust tend to believe in the government’s capacity for effective governance and safety assurance, thereby reducing excessive reactions to potential risks and making them more likely to form rational judgements based on authoritative information. Conversely, groups with low regulatory trust are more inclined to question governmental governance capabilities. Consequently, when confronted with emerging technologies, they are more susceptible to being influenced by diverse or even contradictory information, exhibiting heightened sensitivity to risk perception. Based on this, the following hypothesis is proposed:

**H7a.**  *Compared with individuals with high regulatory trust, those with low regulatory trust are more sensitive to risk perceptions, such that the negative effects of perceived risks on GenAI acceptance are stronger.*

Moreover, regulatory trust not only alleviates the public’s perception of uncertainty regarding risks but also significantly heightens their sensitivity to technological benefits (Gefen et al., 2003). Prior studies also suggest that trust increases the credibility of positive information and strengthens individuals’ willingness to rely on benefit-related judgements when evaluating unfamiliar technologies (Frewer et al., 1996; Siegrist, 2000). Research on institutional trust indicates that when confronted with emerging technologies, the public often regards regulatory trust as an indicator of information source credibility. High levels of institutional trust can elevate societal trust and cooperation (Sønderskov & Dinesen, 2016), making individuals more inclined to adopt positive information conveyed by authoritative bodies. In emerging technology contexts where risks and benefits coexist, regulatory trust is perceived as a manifestation of information source credibility. Within high-trust environments, the public is more inclined to adopt positive technological information conveyed by authoritative bodies, thereby enhancing their cognitive acceptance of the technology’s potential benefits (Frewer et al., 1996). This is also consistent with research showing that trust can reinforce positive evaluations of usefulness and strengthen favorable behavioral intentions toward novel technological systems (Gefen et al., 2003). Consequently, groups exhibiting high regulatory trust are more likely to develop positive perceptions and behavioral intentions when confronted with technological benefits, thereby demonstrating heightened “benefit sensitivity”. Based on this, the following hypothesis is proposed:

**H7b.**  *Compared with individuals with low regulatory trust, those with high regulatory trust are more sensitive to perceived benefits, such that the positive effects of perceived benefits on GenAI acceptance are stronger.*

### 2.2. Research Model

**Figure 1:**
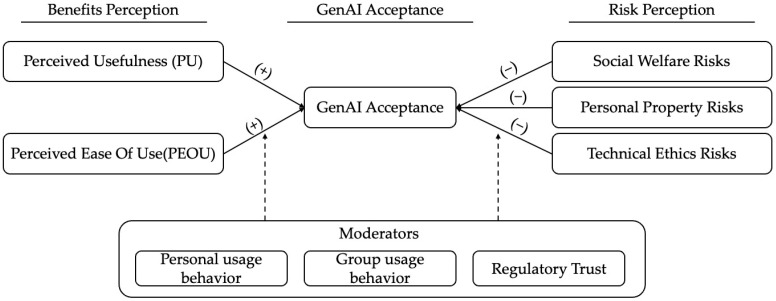
Model of GenAI Public Acceptance.

From the perspective of the benefit dimension within the TAM, the public’s perception of GenAI benefits primarily manifests in two aspects: perceived usefulness and perceived ease of use. To further enrich the theoretical framework, this study incorporates risk perceptions concerning social welfare, personal property, and technological ethics into the analysis, building upon the Emerging Technology Acceptance Model. It explores the impact of different types of risk perceptions and benefit perceptions on public GenAI acceptance, as well as their differentiated mechanisms of action. Furthermore, driven by theoretical refinement and practical considerations, this study introduces three moderating variables: individual usage behavior, group usage behavior, and trust in regulation. Consequently, a theoretical model of public GenAI acceptance is constructed under the dual perception perspective of “benefits-risks” (see Figure 1). This framework aims to systematically elucidate the underlying mechanisms influencing public GenAI acceptance, thereby providing theoretical underpinnings for related technology promotion and governance practices.

## 3. Research Design and Data

### 3.1. Research Design

This study employs a survey-experimental approach to examine public attitudinal responses to perceived GenAI benefits and risks under randomized exposure. Unlike previous research that relies primarily on direct questionnaire surveys of respondents’ attitudes (Siegrist & Cvetkovich, 2000), this design allows respondents to evaluate GenAI within specific contextual scenarios while reducing some confounding influences associated with omitted variables and common-source bias (Antonakis et al., 2010; Podsakoff et al., 2003). Accordingly, compared with conventional questionnaire surveys, survey experiments can provide stronger internal comparability across conditions and support a more cautious analysis of how different risk-framed scenarios are associated with public attitudes toward GenAI.

First, compared to simple questionnaire surveys, survey experiments can more realistically simulate the public’s process of using and accepting GenAI within specific life contexts. Simultaneously, this approach mitigates interference from social desirability bias. Within survey experiments, respondents must make choices and judgements within specific simulated tasks, thereby more authentically reflecting their attitudes and behaviors and enhancing data credibility. Second, by embedding experimental interventions within the framework of social surveys, survey experiments can improve control over potential confounding factors such as individual differences and contextual variation (Gaines et al., 2007; Mutz, 2011). In this sense, the design is helpful for analyzing attitudinal differences across experimental conditions, even though the findings should still be interpreted cautiously as evidence of scenario-based attitudinal responses rather than strong causal effects.

This study designed four distinct questionnaires addressing social welfare loss, personal property loss, technical–ethical conflict, and a control group (see Table 1). Based on these, three parallel survey experiments were constructed to compare outcomes between experimental and control groups, thereby assessing the impact of the intervention. Experiment One focused on macro-level societal consequences of GenAI, including social inequality, collective welfare disruption, and broader information-related risks, and was therefore treated as a social welfare risk scenario. Experiment Two emphasized direct individual-level material harms, including employment insecurity, personal data exposure, and economic disadvantage, and was therefore treated as a personal property loss scenario. Experiment Three focused primarily on norm-based concerns regarding fairness, transparency, accountability, and algorithmic bias, and was therefore treated as a technological–ethical risk scenario. Although some concrete manifestations of GenAI risk may overlap across real-world contexts, each vignette was designed to foreground a different dominant risk emphasis rather than to constitute a fully mutually exclusive risk category. Drawing upon relevant theories, prior research indicates that individual characteristics such as knowledge level, social trust, technological familiarity, gender, and educational attainment may exert interference effects on technology acceptance (Marangunić & Granić, 2015). Consequently, this study incorporates observable variables such as public GenAI usage behavior, regulatory trust, risk preference, alongside gender, educational attainment, and science learning experience as control factors in the questionnaire design. To mitigate potential interference from scenario settings on perceived benefits, the benefit perception questions were placed before the scenario questions in the experiment.

### 3.2. Questionnaire Distribution

The survey component of this research was conducted by a professional questionnaire distribution company. The core of the experimental design lies in effectively mitigating potential endogeneity issues, such as omitted variables, by randomly assigning participants to different scenarios and conditions (Yin et al., 2025). Unlike traditional questionnaires, which typically emphasise sample representativeness and inferences about the population, survey experiments prioritise ensuring the random distribution of intervention variables within the sample. Provided subjects in the experimental and control groups are randomly assigned, this approach effectively safeguards the internal validity of the experimental effect. Therefore, this study requires the survey company to distribute four types of questionnaires using a completely randomised approach within a pre-determined respondent group, ensuring no overlapping samples between the experimental and control groups. Following data collection, randomisation checks were conducted to assess the comparability of the treatment and control groups on key pre-treatment variables. Furthermore, this study employs an online questionnaire format. Compared to offline methods, online surveys enhance response completeness through mandatory questions while facilitating nationwide coverage. This ensures relatively diverse data sources across regions, although it does not eliminate the demographic bias of the online sample. Accordingly, the findings should be interpreted as more reflective of China’s internet-user population than of the national population as a whole.

In the experimental procedure, participants were randomly assigned to either the experimental or control group via an online platform. Participants in the experimental group were randomly assigned to read relevant contextual materials. To ensure information accuracy, all materials were sourced from journal articles. To prevent potential interference from journal source information, specific journal details were deliberately omitted from the experimental materials. Following their reading of the relevant scenario materials, experimental group participants proceeded to complete questions regarding GenAI acceptance. In contrast, control group participants did not read any descriptive materials and directly answered questions about GenAI acceptance. The use of a no-scenario control group is also consistent with prior survey-experimental research on emerging technology acceptance, where treatment groups were exposed to risk-framed materials while the control group received no additional vignette information (e.g., Zhu & Lou, 2024). More broadly, this design follows the logic of randomized survey experiments, in which untreated baseline groups serve as the comparison point for information-based interventions (Gaines et al., 2007; Mutz, 2011). Furthermore, we strictly required the survey company to ensure no overlap between participants from different groups during both the distribution and collection of questionnaires.

### 3.3. Questionnaire Data

The questionnaire comprises two sections: Part One primarily investigates respondents’ perceived benefits, technology usage behavior, trust in regulation, and demographic information; Part Two features a randomised experiment and questions regarding technological attitudes. To facilitate respondent comprehension, the term “GenAI” has been uniformly replaced with “AI tools (such as DeepSeek, Doubao, etc.)” throughout this study (see Table 2 for details). Specifically, we measured public GenAI acceptance through the question: “What is your attitude towards using AI tools (such as DeepSeek, Doubao, etc.)?” This question employed a rating scale from 1 to 9, with higher scores indicating greater public acceptance of the technology.

Moreover, several key constructs in the present study were measured using single-item indicators in order to reduce respondent burden and maintain feasibility within the scenario-based survey design. This study collected a total of 1217 valid questionnaires, with IP addresses covering most regions of China, thereby providing a relatively comprehensive reflection of the national situation. The number of questionnaires for the three experimental groups and the control group were 302, 307, 302, and 306 respectively. Considering the questionnaire’s length and complexity, this study filtered samples with completion times between 30 and 300 seconds to exclude invalid responses due to excessively short or long completion times, thereby ensuring the accuracy of experimental results. Simultaneously, respondents were filtered by age, retaining data from individuals aged 18 to 80. This yielded 251, 244, 253, and 236 valid questionnaires for the three experimental groups and the control group respectively, totalling 984 valid samples.

The core of the experimental survey method lies in utilising randomised grouping to eliminate potential systematic differences between experimental and control groups. This approach effectively addresses endogeneity issues such as omitted variables, thereby improving the internal comparability of the experimental groups. Furthermore, this methodology imposes relatively relaxed assumptions on statistical models; in many instances, ordinary least squares or non-parametric analysis suffice to conduct causal inference, offering both simplicity and high statistical power (Gaines et al., 2007). Following data collection, we conducted randomisation tests. By performing between-group difference tests for each of the three experimental groups against the control group (see Table 3). Overall, the four groups were broadly comparable, suggesting that the random assignment worked reasonably well. At the same time, several significant or near-significant between-group differences remained. In particular, group usage behavior differed significantly in the comparison between the social welfare group and the control group (*p* = 0.023), and gender differed significantly in the technical ethics group (*p* = 0.003). In addition, several variables, including perceived ease of use, age, scientific studies experience, and education, were close to conventional significance thresholds in some comparisons. Some scholars contend that even with fully randomised grouping, demographic imbalances may occur under conditions of limited sample size; such “imbalances” are a natural consequence of randomised designs. Provided that imbalanced variables are controlled for in the analysis, the internal validity of the experiment remains substantially unaffected (Gerber & Green, 2012). Following standard practice in experimental research, we therefore retained these variables as controls in the subsequent regression analyses.

Table 3 also presents the descriptive statistics for key variables in this study. The sample data indicates that the average age of respondents was 31.14 years, with educational attainment predominantly at the college or undergraduate level. Overall, the sample was younger and more highly educated than the national population. In addition, most respondents had a science-based educational background and relatively high levels of regulatory trust. Because the survey was distributed primarily through online channels, the sample may be subject to selection bias related to age, education, and internet access. At the same time, according to data from the China Internet Network Information Centre, China’s internet-user population is itself relatively young and increasingly well educated. Therefore, the present sample may be considered broadly relevant to the demographic profile of Chinese internet users, but it should not be treated as fully representative of the national population. We therefore interpret the findings with appropriate caution and view them as more informative about online public attitudes toward GenAI than about the attitudes of the entire population. To further assess robustness, we conducted age-stratified regression analyses, and the results remained substantively consistent with the main findings.

## 4. Analysis and Discussion

**Figure 2:**
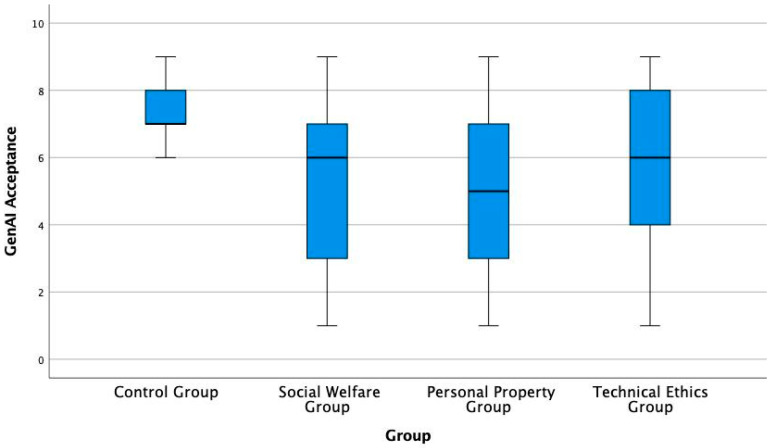
Mean values and 95% confidence intervals for GenAI acceptance in the experimental and control groups.

After confirming that the treatment and control groups were broadly comparable overall, while controlling for several significant or near-significant pre-treatment differences, this study further analysed the core dependent variable—the overall public GenAI acceptance and differences between groups (see Figure 2). The figure indicates that while public GenAI acceptance is generally high at present, the average acceptance levels across all three experimental groups were significantly lower than that of the control group.

To improve comparability across the three experiments, all regression models reported in Section 4.1, Section 4.2 and Section 4.3 use the same set of control variables and include provincial fixed effects. In addition, VIF tests for the three experiments all yielded values below 5, indicating no serious multicollinearity. The discussion below therefore focuses on the substantive differences in how the three risk scenarios shape public GenAI acceptance.

### 4.1. Social Welfare Group

In Experiment 1, respondents in the treatment group were exposed to a scenario emphasizing potential social welfare losses associated with GenAI, whereas the control group received no such scenario. As shown in Table 4, regression findings further demonstrated that public perception of “GenAI causing social welfare losses” significantly reduced acceptance of GenAI. This implies that when the public perceives GenAI as potentially detrimental to overall social welfare, their overall willingness to accept it diminishes significantly, confirming H3. Concurrently, the study found that enhanced PU and PEOU significantly increase public acceptance of GenAI, supporting H1 and H2. This aligns with the fundamental assumptions of the classic TAM (Davis, 1989), further supporting the pivotal role of perceived benefits in GenAI technology acceptance behavior. Moreover, empirical findings reveal that within the social welfare risk dimension, risk perception and perceived benefits jointly influence public willingness to adopt GenAI. This indicates that the relationship between the two is not a linear trade-off but constitutes a complex balancing mechanism, reflecting the multifaceted considerations of the public in high-uncertainty scenarios.

### 4.2. Personal Property Group

Experiment 2 focuses on the personal property loss scenario, in which respondents were exposed to potential individual-level harms such as job displacement, privacy breaches, and material disadvantage. As shown in Table 5, regression results show that public perception of the risk that “GenAI may cause personal property losses” significantly reduced acceptance of GenAI, suggesting greater public caution towards emerging technologies when direct individual interests are threatened, supporting H4. Concurrently, perceived usefulness and perceived ease of use continued to exert significant positive effects on technology acceptance, further supporting H1 and H2. Although PU and PEOU still exert positive effects, the results suggest that risk perception plays a more dominant role in this scenario than in Experiment 1. In other words, compared with broader social-welfare concerns, risks tied to personal property and direct individual loss appear more capable of suppressing public acceptance. This pattern may reflect the greater psychological salience of concrete and personally relevant losses when the public evaluates emerging technologies.

### 4.3. Technical Ethics Group

Experiment 3 examines the technical–ethical risk scenario, in which the treatment group was exposed to concerns related to fairness, privacy, transparency, and algorithmic bias. As reported in Table 6, regression results show that public risk perception regarding “GenAI triggering ethical conflicts” significantly reduced acceptance of the technology, suggesting ethical uncertainty is also a key inhibitory factor in shaping public technological attitudes, supporting H5. Consistent with the previous experiments, PU and PEOU still exerted a significant positive influence on acceptance within the ethical risk scenario, further supporting H1 and H2. However, unlike the personal property scenario, PU and PEOU appear to play a more prominent role in shaping acceptance. This suggests that while the public remains vigilant when confronting technological ethical controversies, they are more inclined to weigh the technology’s value from the perspective of its benefits. Consequently, perceived benefits exert a stronger guiding effect within this dimension.

**Figure 3:**
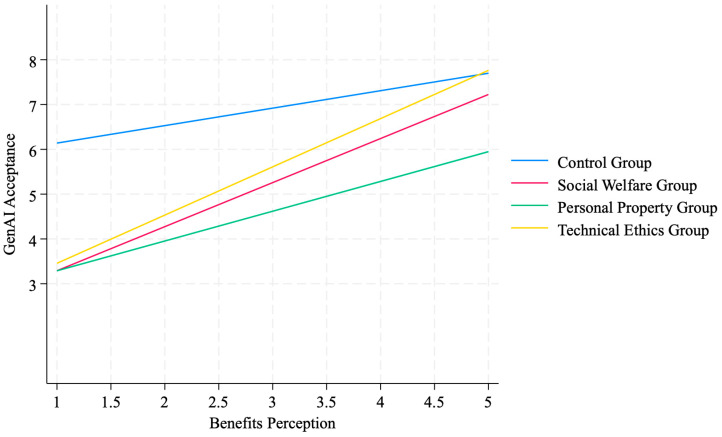
The Marginal Effect of Perceived Returns on GenAI Acceptance Across Different Risk Scenarios.

Figure 3 illustrates the marginal prediction value changes in public GenAI acceptance across different risk scenarios, as influenced by perceived benefits. Findings indicate that within the technological ethics risk group, perceived benefits exert the strongest positive influence on acceptance; this effect is moderate within the social welfare group; whereas in the personal property risk scenario, the positive effect is weakest and partially offset by perceived risks. In summary, the three experiments collectively show differentiated associations between risk perception, perceived benefits, and GenAI acceptance across the three scenarios. They further reveal the differing mechanisms by which distinct types of risk perception influence public attitudes, highlighting the complex and differentiated acceptance mechanisms at play when the public encounters GenAI.

To formally assess whether the treatment effects differ across the three risk scenarios, we estimated a pooled model and conducted pairwise Wald tests comparing the coefficients of the social welfare, personal property, and technical ethics scenarios (see Table 7). The results show that all three risk scenarios significantly reduce public GenAI acceptance relative to the control group. Moreover, the negative effect of the personal property scenario is significantly stronger than that of both the social welfare scenario and the technical ethics scenario. The negative effect of the social welfare scenario is also significantly stronger than that of the technical ethics scenario, although this difference is comparatively smaller. These results indicate that the three risk scenarios exert statistically distinguishable negative effects on public GenAI acceptance.

### 4.4. Testing Heterogeneity in Technology Usage Behavior and Regulatory Trust

Given that emerging technologies are characterised by “radical innovation” and “uncertainty and ambiguity” (Rotolo et al., 2015), public perceptions of the benefits and risks associated with a given technology may vary depending on individuals’ technology usage behavior and levels of trust in regulation. This study therefore further examines the moderating role of technology usage behavior in the relationship between perceived benefits, perceived risks, and public GenAI acceptance (see Table 8, Table 9 and Table 10). Specifically, while Table 8 and Table 9 present subgroup-based heterogeneity results for personal usage behavior and group usage behavior, Table 10 reports formal interaction models to provide a more rigorous test of whether technology usage behavior moderates the effects of perceived benefits and risk-framed scenario exposure on public GenAI acceptance. In these interaction models, Scenario Treat, PU, PEOU, and the corresponding usage-behavior indicators are entered together with their interaction terms, while retaining the same control variables and provincial fixed effects as in the main regressions. In the questionnaire, the item “ How frequently have you used AI tools over the past three months?” measured individual technology usage behavior, while “ How do you observe the use of AI tools among your colleagues/friends?” measured group technology usage behavior. Both are frequency variables. Responses of “Never used” and “1–2 times per week” were grouped as low usage frequency, while “Hardly anyone uses” and “A few people use” were also combined into this category. The remaining options were aggregated into the high usage frequency group.

Descriptive subgroup results in Table 8 and Table 9 suggest that technology usage behavior is associated with heterogeneous responses to GenAI risks and benefits. On the risk side, the negative effects of the scenario treatments are generally stronger among respondents with higher personal or group usage frequency, especially in the social welfare and personal property scenarios. On the benefit side, PU and PEOU remain positively associated with GenAI acceptance across most subgroup models, but the differences between low- and high-usage groups are less consistent.

To test whether these subgroup patterns are statistically meaningful, Table 10 reports formal interaction models for personal usage behavior and group usage behavior. The results indicate that the moderating role of technology usage behavior is selective rather than universal. At the individual level, the interaction between Scenario Treat and High Personal Usage is significantly negative in the social welfare scenario, and the joint interaction test is also significant, indicating stronger risk sensitivity among frequent users in this context. In the technical ethics scenario, the joint interaction test is marginally significant, whereas in the personal property scenario the evidence is weaker. At the group level, the interaction between Scenario Treat and High Group Usage is significantly negative only in the social welfare scenario, while the joint interaction tests are not significant across the three models, suggesting comparatively limited formal evidence of moderation. On the benefit side, PU remains positive and significant across all six models, but the interaction terms involving PU are consistently insignificant. Significant benefit-side moderation appears only for PEOU × HPU in selected scenarios.

Taken together, the evidence suggests selective rather than universal moderation. Technology usage behavior appears to strengthen public sensitivity to risk-framed scenario exposure more clearly than it strengthens sensitivity to perceived benefits, and the formal evidence is stronger for personal usage behavior than for group usage behavior. This pattern is broadly consistent with Prospect Theory, which suggests that individuals tend to be more sensitive to losses than to gains (Kahneman & Tversky, 2012). It may also reflect a habituation effect, whereby the benefits of technology use become normalized with repeated exposure, while risks remain more salient when users encounter concrete problems in practice (Limayem et al., 2007). Therefore, H6a and H6b are only partially supported. Overall, these findings suggest that the moderating role of technology usage behavior operates more strongly on the risk side than on the benefit side, which is broadly consistent with existing findings on emerging technology acceptance (Zhu & Lou, 2024).

Given the significance of government regulation in shaping public perceptions of technology, this study further analyzes how levels of regulatory trust influence perceptions of the benefits and risks associated with GenAI (see Table 11 and Table 12). In the literature, regulatory trust is generally understood as the degree to which the public recognizes regulators’ technical capabilities, motivations, and institutional development (Bratspies, 2009). In the present study, however, regulatory trust is operationalized in a relatively narrow way. Specifically, regulatory trust is operationalized through respondents’ answers to the question of whether they feel comfortable entrusting AI-related usage data to the central government. This measurement approach was informed by related prior research on public risk perception and emerging technology acceptance (Zhu & Lou, 2024). The item captures one concrete dimension of trust in regulatory authority, namely confidence in state data governance and regulatory oversight. Therefore, the moderating results reported in this study should be interpreted as reflecting a specific form of trust in regulatory oversight, rather than the full multidimensional construct of regulatory trust discussed in the broader literature, including dimensions such as competence, integrity, and benevolence. Accordingly, the moderation patterns reported below should be understood as pertaining only to this narrow operationalization, rather than as a comprehensive test of regulatory trust as a whole. The questionnaire employed a five-point response scale: “Very confident,” “Confident,” “Neutral,” “Not very confident,” and “Very not confident.” Respondents were categorized into “high trust” and “low trust” groups based on their scores, with scores of 1–2 assigned to the low trust group and scores of 3–5 to the high trust group.

According to the descriptive subgroup results in Table 11, the low regulatory trust group appears to be more sensitive to risk perceptions than the high regulatory trust group. Across the three risk scenarios, perceived social welfare loss, personal property loss, and technical-ethical conflict are all associated with lower GenAI acceptance, and these negative effects are generally stronger in the low-trust group. By contrast, the high-trust group shows a more stable and consistently positive response to perceived usefulness and perceived ease of use. These subgroup patterns are broadly consistent with H7a and provide only limited descriptive indication for H7b. At the same time, these results should be interpreted with caution, especially in the personal property scenario, where the low-trust subgroup has a very small sample size (N = 19) and displays an unusual coefficient pattern. Accordingly, these subgroup estimates are treated as supplementary rather than decisive evidence, are reported primarily for completeness, and are not used as a key basis for evaluating H7a or H7b.

To provide a more rigorous test of moderation, Table 12 further reports formal interaction models for regulatory trust. The results suggest that the moderating role of regulatory trust is limited rather than universal. On the risk side, the interaction between Scenario Treat and High regulatory trust is positive and statistically significant only in the social welfare scenario, indicating that the negative effect of risk-framed exposure is weaker among respondents with higher confidence in regulatory oversight in this context. However, the corresponding interaction terms in the personal property and technical ethics scenarios are not significant, and the joint interaction tests are not significant across all three models. On the benefit side, neither PU × High regulatory trust nor PEOU × High regulatory trust reaches statistical significance in any of the three scenarios. This suggests that formal interaction evidence for strengthened benefit sensitivity under high regulatory trust remains weak.

The findings of this study are broadly consistent with previous research on the relationship between regulatory trust and technology acceptance (Chawla et al., 2024; Yaseen & Al-Amarneh, 2025; Zahlan et al., 2023). However, the present results should be interpreted cautiously. The descriptive subgroup results suggest that respondents with lower regulatory trust may be more sensitive to risk-framed information, but the formal interaction models provide only limited support for this pattern. In particular, risk-side moderation is statistically significant only in the social welfare scenario, and the joint interaction tests are not significant across the three models. On the benefit side, the interaction terms involving perceived usefulness and perceived ease of use are not statistically significant, indicating that evidence for stronger benefit sensitivity under higher regulatory trust remains weak. Therefore, any implication that regulatory trust improves the reception of benefit-related information should be treated as tentative. Taken together, the evidence provides limited but relatively more consistent support for H7a and only weak and non-robust support for H7b. In other words, regulatory trust appears more clearly related to differential sensitivity to risk than to systematically stronger sensitivity to perceived benefits. Accordingly, the moderating role of regulatory trust should be interpreted with caution, and the very small low-trust subgroup in the personal property scenario is not treated as a key source of support for these hypotheses.

## 5. Conclusions

### 5.1. Conclusions and Contributions

First, based on three parallel survey experiments, this study shows that perceived benefits and perceived risks jointly shape public GenAI acceptance. Perceived usefulness and perceived ease of use consistently exert positive effects on acceptance, whereas the three risk scenarios—social welfare loss, personal property loss, and technical–ethical conflict—all significantly reduce acceptance. At the same time, the results reveal meaningful cross-scenario variation. Personal property loss produces the strongest negative effect, social welfare loss occupies an intermediate position, and technical–ethical conflict shows the weakest negative effect among the three. In addition, the heterogeneity analyses suggest that technology usage behavior and regulatory trust condition these relationships in selective rather than universal ways. Overall, the evidence indicates that within this online sample of Chinese internet users, risk–benefit evaluations of GenAI are differentiated across both risk contexts and user characteristics.

Table 13 summarizes the overall hypothesis-testing results. In general, H1–H5 receive empirical support, indicating that perceived usefulness and perceived ease of use are positively associated with GenAI acceptance, whereas the three types of risk-framed perceptions are associated with lower acceptance. The moderating hypotheses receive more differentiated support: H6a and H6b are partially supported, while H7a receives limited support with caution and H7b is not robustly supported, given the limited and asymmetric evidence for regulatory-trust moderation.

At the theoretical level, this study’s main contributions are reflected in the following three aspects: First, building upon the Emerging Technology Acceptance Model, this study incorporates “benefit perception” and “risk perception” into the analytical framework, thereby expanding the model’s explanatory power regarding public behavior in emerging technology contexts. Furthermore, by treating risk perception as a core variable influencing acceptance willingness, it constructs a “benefit–risk” dual-path mechanism, enriching the endogenous structure of technology acceptance theory. Second, by designing three risk scenarios—social welfare, personal property, and technological ethics—this study identifies distinct adoption pathways under different risk types. It proposes a mechanism classification of “joint influence—risk dominance—benefit dominance,” deepening understanding of risk heterogeneity and enhancing the contextual explanatory power of technology acceptance research. Third, by introducing two key variables—technology usage behavior and regulatory trust—this study suggests that technology usage behavior and regulatory trust may play meaningful moderating roles across different groups. These findings provide preliminary empirical evidence for incorporating such factors into models of public acceptance of GenAI, while the interpretation of these theoretical implications should remain cautious given the single-item measurement of several key constructs.

In summary, this study deepens the theoretical explanation of emerging technology acceptance behavior from a dual “benefit–risk” perspective, revealing the heterogeneous impact of different risk types and user characteristics on acceptance pathways. It offers empirical evidence that helps illuminate how members of the public evaluate emerging technologies under conditions of uncertainty. It is important to note that public technology acceptance does not always align with objective risk levels. Taking ChatGPT as an example, despite no significant rise in unemployment rates in current macroeconomic data, “fear of unemployment” in public discourse has markedly increased. This phenomenon of “perceived risk preceding actual risk” indicates that policymakers cannot rely solely on objective data to assess societal resistance to technology adoption. As revealed by relevant research (Slovic, 1993), public behavior is more grounded in subjective risk perceptions than objective statistical outcomes. The findings also have tentative implications for GenAI governance and public communication. Given that public evaluations of benefits and risks vary across different scenarios, more context-sensitive communication strategies may be useful. In contexts where ethical risks appear less dominant and perceived utility remains salient, highlighting concrete use cases and practical benefits may help foster more informed acceptance. By contrast, in contexts where personal property loss is more salient, strengthening user data protection, accountability mechanisms, and corporate risk management may help alleviate public concern. In addition, for groups that appear more sensitive to perceived risks, such as frequent users or those expressing lower confidence in regulatory oversight, more targeted risk communication and public education may improve mutual understanding. These implications should be interpreted cautiously in light of the study’s design, measurement limitations, and the restricted external validity of the online sample.

Beyond these substantive findings, the results should also be understood within their broader socio-cultural context. Another contextual factor that may help explain the findings is the role of cultural norms. Because this study was conducted in China, public responses to GenAI may have been shaped not only by perceived benefits and risks at the individual level, but also by broader social expectations concerning collective welfare, social order, and trust in public authority. In particular, concerns about social welfare losses and technological–ethical conflicts may be interpreted through culturally embedded norms regarding fairness, responsibility, and social stability. While the present study does not directly measure cultural norms, we acknowledge that they may provide an important contextual background for understanding public acceptance of emerging technologies. Future research could examine this factor more explicitly in cross-cultural or comparative settings.

### 5.2. Limitations of the Study

Despite its theoretical and empirical contributions, this study has several limitations. First, the experimental design is subject to certain identification constraints. Because the treatment groups were exposed to risk-framed scenario materials whereas the control group received no additional vignette, the design cannot fully disentangle the effect of specific risk content from the more general effect of scenario exposure itself. Moreover, the study did not include direct post-treatment manipulation checks, making it impossible to verify with certainty whether each scenario exclusively activated the intended risk dimension. Accordingly, the findings should be interpreted as evidence of attitudinal responses to different types of risk-framed information, rather than as strong mechanism-based causal estimates.

Second, the data were collected primarily through online questionnaire experiments, and the sample was skewed toward younger and more highly educated respondents. Although the findings are informative for understanding attitudes among Chinese internet users, their generalizability to the broader national population remains limited. Third, several core variables, including perceived usefulness, risk perception, and technology acceptance, were measured using single-item self-reported indicators. As a result, the study may still be subject to common-source bias and social desirability effects, even though the experimental design and control variables help mitigate some of these concerns. In addition, the use of single-item measures may have limited the reliability and construct validity of these variables, and the study’s theoretical implications should therefore be interpreted with appropriate caution.

Fourth, the regulatory trust variable was measured solely through the single item “whether respondents feel comfortable entrusting data from AI tools to the central government.” While this item holds some representativeness in related research (Zhu & Lou, 2024), it fails to capture the multidimensional structure of trust, such as competence, integrity, and benevolence, resulting in measurement oversimplification. Accordingly, the moderation findings related to regulatory trust should be interpreted as preliminary and narrowly scoped, rather than as a comprehensive assessment of institutional trust in a broader sense. Finally, survey experiments primarily capture attitudinal responses under specific scenarios, which may not fully correspond to actual and sustained technology-use behavior in real-world settings. Public responses to GenAI are likely to be shaped by a broader set of influences, including media exposure, social interaction, and practical experience.

### 5.3. Future Directions

Future research can extend the present study in at least three directions. First, methodological rigor could be strengthened by introducing neutral-information control conditions, direct post-treatment manipulation checks, and more diverse forms of evidence, such as multi-source data, behavioral usage records, platform-generated data, or mixed-methods approaches including qualitative follow-up interviews. In addition, future work should adopt more structured and multidimensional measures of regulatory trust in order to capture its distinct components more accurately.

Second, future studies could enhance external validity by drawing on more representative samples and by comparing different demographic, regional, and national contexts. Cross-regional or cross-national comparative designs would help clarify whether the observed patterns are specific to Chinese internet users or extend more broadly across social settings. The theoretical framework could also be extended by incorporating additional factors beyond the current model, such as trust in AI itself and external informational influences including media coverage, scandal salience, or GenAI hype effects.

Third, subsequent research could move beyond one-time attitudinal responses by examining more specific application domains, such as education, public administration, and healthcare, and by employing longitudinal designs, panel surveys, or embedded field experiments. Such designs would help reveal how public attitudes toward GenAI evolve over time and how perceived benefits and risks translate into real-world technology adoption. Validation could also be strengthened through cumulative comparison with other GenAI studies, more explicit reporting of effect sizes, and experimental tests of risk-mitigation measures such as transparency labels or disclosure cues.

## Figures and Tables

**Table 1:** Investigative Experiment Reading Materials.

Investigative Experiment	Reading Materials	Source
Experiment 1 Social Welfare Group	GenAI (such as DeepSeek and ChatGPT) is reshaping social structures at an unprecedented pace, yet the risks it poses are equally significant: Regarding employment, the International Monetary Fund (IMF) indicates that approximately 40% of global jobs face the risk of being replaced by AI, with the proportion reaching as high as 60% in developed countries, potentially exacerbating global inequality. Regarding information security, a 2024 UK survey revealed that 20% of companies experienced data breaches due to employees using AI chatbots, exposing weaknesses in internal security management. Furthermore, AI-generated misinformation has become a major short-term threat to the global economy. The World Economic Forum warned in 2024 that AI-driven disinformation could disrupt electoral processes and trigger social unrest.	(Kristalina, 2024; Torkington, 2024)
Experiment 2 Personal property Group	GenAI (such as DeepSeek and ChatGPT) is rapidly reshaping personal interests: In job displacement, the translation industry saw a 37.2% plunge in revenue, while financial analyst positions face elimination as AI boosts decision-making efficiency by 40%. Regarding privacy risks, Samsung employees caused three leaks of trade secrets by using ChatGPT, and open-source library vulnerabilities in 2023 exposed user payment information. The harm from misinformation is particularly severe, with AI-driven disinformation factories churning out 100,000 false content pieces daily. By 2025, rumors like “intelligent driving fatalities” caused 200 million yuan in economic losses. Technological monopolies exacerbate class divisions: while high-skill positions saw 20% wage growth, low-skill workers experienced a 35% income plunge, pushing the global Gini coefficient up by 0.15 points due to AI. These figures reveal that AI’s technological dividends come at the cost of ordinary people’s job security, privacy rights, and economic fairness.	(Brundage et al., 2018; Nartey, 2025; Sholler & MacInnes, 2024; Yao et al., 2024)
Experiment 3 Technical Ethics Group	GenAI (such as DeepSeek and ChatGPT) poses multiple risks in terms of technological ethics. Regarding job displacement, a report by the McKinsey Global Institute indicates that by 2030, approximately 14% of the global workforce (about 375 million people) may be forced to change careers or even lose their jobs due to AI technology. Regarding privacy and security, a March 2025 report highlighted that hackers exploit malicious code within open-source AI models to steal information or tamper with outputs, posing significant risks to businesses. Concerning algorithmic bias, studies reveal that AI models may unintentionally discriminate against job seekers of specific genders, races, or ages, depriving them of fair employment opportunities.	(McKinsey & Company, n.d.; Haltaufderheide & Ranisch, 2024; Yao et al., 2024)

Note: As this study was conducted in China, all questionnaires distributed were in Chinese. To facilitate reader comprehension, we have translated the Chinese reading materials into English. The three experimental vignettes were designed to emphasize different dominant types of GenAI-related risk—social welfare, personal property, and technical ethics—rather than to eliminate all possible overlap in concrete real-world risk manifestations.

**Table 2:** Key Variable Descriptions.

Key Variable	Question Corresponding to the Questionnaire
Adoption of GenAI	Your stance on using AI tools such as DeepSeek, Doubao, Wenxin Yiyan, Tencent Cloud Bao, etc.
Perceived usefulness	To what extent do you believe AI tools enhance your work efficiency?
Perceived ease of use	How challenging do you find learning and using AI tools?
Personal usage behavior	How frequently have you used AI tools over the past three months?
Group usage behavior	How do you observe the use of AI tools among your colleagues/friends?
Level of risk aversion	If you were presented with two lottery tickets to choose from, with the first offering a 100% chance of winning ¥4000 and the second a 50% chance of winning ¥8000 and a 50% chance of winning nothing, which would you select?
Regulatory Trust	Would you feel comfortable entrusting the data logs from your use of AI tools to the central government for safekeeping?
Scientific Studies Experience	During your university (or secondary school) studies, did you lean towards arts subjects or science subjects?

Note: As this study was conducted in China, all questionnaires distributed were in Chinese. To facilitate reader comprehension, we have translated the Chinese reading materials into English. Moreover, all questionnaire items were derived from journal literature and appropriately revised.

**Table 3:** Mean values of variables and randomisation tests for experimental and control groups.

Variables	All Sample	Control Group	Social Welfare Group		Personal Property Group		Technical Ethics Group	
	Mean	Mean	Mean	*p*-Value	Mean	*p*-Value	Mean	*p*-Value
PU	3.61	4.06	3.68	0.273	4.02	0.255	3.58	0.233
PEOU	3.48	3.86	3.55	0.051	3.79	0.074	3.48	0.088
Personal usage behavior	3.07	2.60	3.08	0.790	2.59	0.805	3.08	0.813
Group usage behavior	2.94	2.86	2.96	0.023	2.88	0.634	2.90	0.288
Level of risk aversion	−0.01	−0.08	−0.05	0.447	−0.02	0.177	−0.04	0.395
Regulatory Trust	3.58	4.01	3.59	0.482	3.99	0.489	3.58	0.486
Gender	0.52	0.55	0.55	0.911	0.56	0.815	0.48	0.003
Age	31.14	30.03	31.90	0.053	28.86	0.066	31.56	0.063
Scientific Studies Experience	1.98	2.12	2.06	0.160	2.00	0.086	2.01	0.073
Education	2.01	1.98	2.00	0.448	1.97	0.506	2.03	0.050

Note: The reported *p*-values are based on between-group difference tests comparing each experimental group with the control group.

**Table 4:** Social Welfare Group and Control group.

	Variables	Coef.	Robust Standard Errors
Perception of Social Welfare Risks	Scenario Treat (Social welfare loss)	−0.826 ***	0.250
Benefits Perception	PU	0.489 ***	0.0733
	PEOU	0.324 ***	0.0733
Control variables	Personal usage behavior	−0.00968	0.0808
	Group usage behavior	0.0263	0.0749
	Level of risk aversion	−0.0803	0.0668
	Regulatory Trust	0.655 ***	0.0966
	Gender	−0.0104	0.131
	Age	−0.00629	0.00972
	Scientific Studies Experience	−0.0883	0.0741
	Education	0.125	0.178
C	Constant	1.541 **	0.679
	N	487	
	R^2^	0.532	
	Province FE	YES	

Note: *** *p* < 0.01, ** *p* < 0.05, Mean VIF = 1.31.

**Table 5:** Personal Property Group and Control group.

	Variables	Coef.	Robust Standard Errors
Perception of Personal Property Risks	Scenario Treat (Personal Property loss)	−1.958 ***	0.157
Benefits Perception	PU	0.406 ***	0.142
	PEOU	0.295 ***	0.0625
Control variables	Personal usage behavior	0.250 *	0.121
	Group usage behavior	0.0230	0.109
	Level of risk aversion	−0.0389	0.0583
	Regulatory Trust	0.434 **	0.201
	Gender	0.157	0.201
	Age	−0.00422	0.00789
	Scientific Studies Experience	−0.0613	0.0819
	Education	0.0295	0.114
C	Constant	2.220 ***	0.520
	N	480	
	R^2^	0.384	
	Province FE	YES	

Note: *** *p* < 0.01, ** *p* < 0.05, * *p* < 0.1, Mean VIF = 1.17.

**Table 6:** Technical Ethics Group and Control group.

	Variables	Coef.	Robust Standard Errors
Perception of Technical Ethics Risks	Scenario Treat (Technical–ethical Conflict)	−0.555 **	0.220
Benefits Perception	PU	0.513 ***	0.0975
	PEOU	0.413 ***	0.0847
Control variables	Personal usage behavior	0.0514	0.0727
	Group usage behavior	0.162 **	0.0722
	Level of risk aversion	0.0517	0.0511
	Regulatory Trust	0.573 ***	0.106
	Gender	0.239	0.170
	Age	−0.00569	0.0116
	Scientific Studies Experience	−0.0273	0.0781
	Education	0.259 **	0.116
C	Constant	0.356	0.465
	N	489	
	R^2^	0.582	
	Province FE	YES	

Note: *** *p* < 0.01, ** *p* < 0.05, Mean VIF = 1.31.

**Table 7:** Pooled Model and Coefficient-Comparison Tests Across Risk Scenarios.

Variables	Pooled Model
Social welfare	−1.038 *** (0.186)
Personal property	−1.976 *** (0.151)
Technical ethics	−0.711 *** (0.178)
PU	0.493 *** (0.069)
PEOU	0.414 *** (0.059)
Constant	1.252 * (0.645)
Observations	970
R-squared	0.491
Controls	YES
Province FE	YES
*p* (Social welfare = Personal property)	<0.01
*p* (Social welfare = Technical ethics)	0.0496
*p* (Personal property = Technical ethics)	<0.01

Note: Robust standard errors in parentheses. *** *p* < 0.01, * *p* < 0.1. Controls include personal usage behavior, group usage behavior, level of risk aversion, regulatory trust, gender, age, scientific studies experience, and education; province fixed effects are included in the model.

**Table 8:** Testing for Heterogeneity in Personal Usage Behavior.

	Personal Usage Behavior						
		Low	High	Low	High	Low	High
Risk Perception	Social Welfare	−0.208 (0.283)	−1.154 *** (0.358)				
	Personal property			−1.970 *** (0.153)	−1.812 *** (0.227)		
	Technical Ethics					−0.904 * (0.455)	−0.573 ** (0.262)
Benefits Perception	PU	0.423 ** (0.154)	0.577 *** (0.0903)	0.349 (0.210)	0.358 ** (0.155)	0.561 ** (0.201)	0.520 *** (0.124)
	PEOU	0.318 * (0.177)	0.305 *** (0.0928)	0.112 (0.119)	0.519 *** (0.147)	0.229 * (0.134)	0.511 *** (0.0904)
	N	163	324	251	229	165	324
	R^2^	0.579	0.534	0.369	0.437	0.568	0.598
	Province FE	YES	YES	YES	YES	YES	YES

Note: Robust standard errors in parentheses. *** *p* < 0.01, ** *p* < 0.05, * *p* < 0.1. The regression results have been simplified to exclude other variables, presenting only the risk perception and benefit perception dimensions.

**Table 9:** Testing for Heterogeneity in Group Usage Behavior.

	Group Usage Behavior						
		Low	High	Low	High	Low	High
Risk Perception	Social Welfare	−0.259 (0.397)	−1.023 *** (0.302)				
	Personal property			−1.502 *** (0.311)	−2.231 *** (0.269)		
	Technical Ethics					−0.409 (0.332)	−0.488 * (0.279)
Benefits Perception	PU	0.335 ** (0.142)	0.499 *** (0.115)	0.711 *** (0.235)	0.184 (0.119)	0.589 *** (0.165)	0.477 *** (0.124)
	PEOU	0.519 *** (0.179)	0.279 *** (0.0969)	0.300 *** (0.101)	0.336 *** (0.0627)	0.407 *** (0.135)	0.423 *** (0.0961)
	N	159	328	184	296	168	321
	R^2^	0.636	0.507	0.359	0.438	0.647	0.559
	Province FE	YES	YES	YES	YES	YES	YES

Note: Robust standard errors in parentheses. *** *p* < 0.01, ** *p* < 0.05, * *p* < 0.1. The regression results have been simplified to exclude other variables, presenting only the risk perception and benefit perception dimensions.

**Table 10:** Formal Interaction Tests for Technology Usage Behavior.

Variables	Personal Usage Behavior			Group Usage Behavior		
	Social Welfare	Personal Property	Technical Ethics	Social Welfare	Personal Property	Technical Ethics
Scenario Treat	−0.083 (0.321)	−2.104 *** (0.138)	−0.507 (0.326)	−0.241 (0.396)	−1.572 *** (0.260)	−0.422 (0.268)
PU	0.469 *** (0.119)	0.381 * (0.183)	0.553 *** (0.137)	0.417 *** (0.126)	0.575 ** (0.259)	0.602 *** (0.133)
PEOU	0.416 *** (0.144)	0.129 (0.111)	0.241 ** (0.109)	0.530 *** (0.178)	0.260 ** (0.105)	0.475 *** (0.122)
High Personal Usage	0.408 (0.738)	−1.789 * (1.009)	−0.799 (0.789)			
ST × HPU	−0.925 ** (0.395)	0.309 (0.205)	0.024 (0.351)			
PU × HPU	0.050 (0.139)	0.102 (0.177)	−0.055 (0.163)			
PEOU × HPU	−0.125 (0.169)	0.386 * (0.206)	0.252 ** (0.098)			
High Group Usage				1.119 (0.659)	1.088 (0.825)	1.118 (0.673)
ST × HGU				−0.825 ** (0.339)	−0.613 (0.458)	−0.263 (0.243)
PU × HGU				0.062 (0.175)	−0.274 (0.222)	−0.134 (0.165)
PEOU × HGU				−0.272 (0.220)	0.075 (0.097)	−0.077 (0.145)
Constant	1.285 (0.958)	3.444 *** (0.737)	1.004 (0.661)	0.882 (0.735)	1.541 * (0.823)	0.034 (0.683)
Observations	487	480	489	487	480	489
R^2^	0.541	0.388	0.586	0.540	0.392	0.582
Joint interaction *p*-value	0.0447	0.201	0.0644	0.143	0.613	0.585
Controls	YES	YES	YES	YES	YES	YES
Province FE	YES	YES	YES	YES	YES	YES

Notes: Robust standard errors are reported in parentheses. *** *p* < 0.01, ** *p* < 0.05, * *p* < 0.1. Controls include Level of risk aversion, Regulatory Trust, Gender, age, Scientific Studies Experience, and Education; province fixed effects are included in all models.

**Table 11:** Testing for Heterogeneity in Regulatory Trust.

	Regulatory Trust						
		Low	High	Low	High	Low	High
Risk Perception	Social Welfare	−3.392 *** (1.192)	−0.557 ** (0.199)				
	Personal property			−8.275 *** (2.049)	−1.955 *** (0.178)		
	Technical Ethics					−2.620 ** (1.202)	−0.387 (0.273)
Benefits Perception	PU	0.210 (0.194)	0.382 *** (0.108)	−4.264 *** (1.016)	0.360 *** (0.0928)	0.335 (0.310)	0.401 *** (0.0914)
	PEOU	−0.00665 (0.302)	0.141 * (0.0770)	3.719 *** (0.786)	0.305 *** (0.0805)	0.276 (0.286)	0.294 *** (0.0898)
	N	81	406	19	461	85	404
	R^2^	0.353	0.121	0.755	0.375	0.431	0.177
	Province FE	YES	YES	YES	YES	YES	YES

Note: Robust standard errors in parentheses. *** *p* < 0.01, ** *p* < 0.05, * *p* < 0.1. The regression results have been simplified to exclude other variables, presenting only the risk perception and benefit perception dimensions.

**Table 12:** Formal Interaction Tests for Regulatory Trust.

Variables	Social Welfare	Personal Property	Technical Ethics
Scenario Treat	−3.244 ** (1.383)	−2.262 ** (1.050)	−2.742 * (1.343)
PU	0.160 (0.177)	0.436 (0.458)	0.325 (0.286)
PEOU	0.185 (0.345)	0.446 (0.399)	0.319 (0.199)
High regulatory trust	0.147 (1.519)	−0.066 (2.605)	0.126 (1.839)
Scenario Treat × High regulatory trust	2.524 * (1.328)	0.316 (1.142)	2.268 (1.444)
PU × High regulatory trust	0.268 (0.200)	0.156 (0.457)	0.093 (0.307)
PEOU × High regulatory trust	−0.070 (0.375)	−0.105 (0.452)	−0.033 (0.202)
Constant	4.742 *** (1.460)	3.024 (2.220)	3.14 (1.755)
Observations	487	480	489
R^2^	0.565	0.366	0.603
Joint interaction *p*-value	0.271	0.975	0.200
Controls	YES	YES	YES
Province FE	YES	YES	YES

Notes. Robust standard errors are reported in parentheses. *** *p* < 0.01, ** *p* < 0.05, * *p* < 0.1. Controls include personal usage behavior, group usage behavior, Level of risk aversion, Regulatory Trust, Gender, age, Scientific Studies Experience, and Education; province fixed effects are included in all models.

**Table 13:** Summary of Hypothesis Testing Results.

Hypothesis	Expected Relationship	Empirical Support
H1	PU → Acceptance (+)	Supported
H2	PEOU → Acceptance (+)	Supported
H3	Risk perception of social welfare losses → Acceptance (−)	Supported
H4	Risk perception of personal property loss → Acceptance (−)	Supported
H5	Risk perception of ethical conflicts → Acceptance (−)	Supported
H6a	Personal usage behavior moderates	Partially supported
H6b	Group usage behavior moderates	Partially supported
H7a	Low regulatory trust strengthens risk sensitivity	Limited support with caution
H7b	High regulatory trust strengthens benefit sensitivity	Weak evidence; not robustly supported

## Data Availability

Data available on request from the authors.
